# Cytoprotective Mechanisms in Fatty Liver Preservation against Cold Ischemia Injury: A Comparison between IGL-1 and HTK

**DOI:** 10.3390/ijms19020348

**Published:** 2018-01-24

**Authors:** Arnau Panisello-Roselló, Eva Verde, Alexandre Lopez, Marta Flores, Emma Folch-Puy, Anabela Rolo, Carlos Palmeira, Georgina Hotter, Teresa Carbonell, René Adam, Joan Roselló-Catafau

**Affiliations:** 1 Experimental Hepatic Ischemia-Reperfusion Unit, Institut d’Investigacions Biomèdiques de Barcelona (IIBB), Spanish National Research Council (CSIC), 08036 Barcelona, Catalonia, Spain; arnau.panisello.rosello@gmail.com (A.P.-R.); emma.folch@iibb.csic.es (E.F.-P.); ghcbam@iibb.csic.es (G.H.); 2 Department of Cell Biology, Physiology and Immunology, Universitat de Barcelona, Barcelona, 08028 Catalonia, Spain; e.vaaverde@gmail.com (E.V.); mfloreso10@alumnes.ub.edu (M.F.); tcarbonell@ub.edu (T.C.); 3 Centre Hépato-Biliaire, AP-PH, Hôpital Paul Brousse, 94800 Villejuif, France; alexandregl.lopez@gmail.com (A.L.); rene.adam@aphp.fr (R.A.); 4 Centro de Investigación Biomédica en Red de Enfermedades Hepáticas y Digestivas (CIBEREHD), Institut d’Investigacions Biomèdiques August Pi i Sunyer (IDIBAPS), 08036 Barcelona, Catalonia, Spain; 5 Center for Neuroscience and Cell Biology, Universidade Coimbra, 3000-370 Coimbra, Portugal; anpiro@ci.uc.pt (A.R.); palmeira@uc.pt (C.P.)

**Keywords:** cold ischemia injury, liver, IGL-1 solution, HTK solution, mTOR, autophagy and apoptosis

## Abstract

Institute Goeorges Lopez 1 (IGL-1) and Histidine-Tryptophan-Ketoglutarate (HTK) preservation solutions are regularly used in clinical for liver transplantation besides University of Wisconsin (UW) solution and Celsior. Several clinical trials and experimental works have been carried out comparing all the solutions, however the comparative IGL-1 and HTK appraisals are poor; especially when they deal with the underlying protection mechanisms of the fatty liver graft during cold storage. Fatty livers from male obese Zücker rats were conserved for 24 h at 4 °C in IGL-1 or HTK preservation solutions. After organ recovery and rinsing of fatty liver grafts with Ringer Lactate solution, we measured the changes in mechanistic target of rapamycin (mTOR) signaling activation, liver autophagy markers (Beclin-1, Beclin-2, LC3B and ATG7) and apoptotic markers (caspase 3, caspase 9 and TUNEL). These determinations were correlated with the prevention of liver injury (aspartate and alanine aminostransferase (AST/ALT), histology) and mitochondrial damage (glutamate dehydrogenase (GLDH) and confocal microscopy findings). Liver grafts preserved in IGL-1 solution showed a marked reduction on p-TOR/mTOR ratio when compared to HTK. This was concomitant with significant increased cyto-protective autophagy and prevention of liver apoptosis, including inflammatory cytokines such as HMGB1. Together, our results revealed that IGL-1 preservation solution better protected fatty liver grafts against cold ischemia damage than HTK solution. IGL-1 protection was associated with a reduced liver damage, higher induced autophagy and decreased apoptosis. All these effects would contribute to limit the subsequent extension of reperfusion injury after graft revascularization in liver transplantation procedures.

## 1. Introduction

The only curative option for patients suffering from end-stage liver disease remains transplantation. Currently, the number of patients requiring transplants is growing up as well as the needs of available organs for liver transplantation. This fact has obliged physicians to consider the use of marginal livers, such as steatotic ones, although it is well recognized that they increase the primary graft failure apparition after transplantation [1,2]. Besides the quality of the graft to be transplanted, the selection of an organ preservation solution is also considered crucial for the graft viability. This is of particular importance when steatosis is present because it is well know that fatty liver grafts are more vulnerable against ischemia reperfusion injury (IRI) contributing thus to primary liver graft failure [3].

Keeping liver grafts in preservation solutions is a mandatory step when cadaveric livers are used for transplantation purposes. During cold storage, liver grafts are deprived of oxygen and nutrients, leading to the accumulation of metabolic waste. This is concomitant with a significant ATP breakdown and alteration of ionic homeostasis, which in turns increases the acidosis and finally leads to organ failure [4]. The selection of organ preservation solutions is crucial to better protect the liver graft against ischemic insult during organ cold storage being this fact determinant to modulate the subsequent deleterious effects and extension of reperfusion injury after graft revascularization in liver transplantation.

University Wisconsin (UW), Celsior, histidine-tryptophane-ketoglutarate (HTK) and more recently, Institut Georges Lopez-1 (IGL-1) are regularly used in liver transplantation [5]. Briefly, HTK is a cardioplegic solution based on the presence of histidine which contributes to maintain acid basic balance during cold ischemia; as well as the substrates tryptophan and alfa-ketoglutarate [6,7]. Moreover, HTK and IGL-1 solutions contain lower sodium and potassium ion concentrations and lower viscosity compared to UW solution, but show different substrates to anaerobic glycolysis. On the other hand, the cellular protection afforded by UW (the gold standard solution in liver transplantation) and IGL-1 is due, in part, to the presence of oncotic agents in both solutions [8]. This is the case of hydroxyethyl starch (HES) for UW and polyethylene glycol 35 (PEG35) for IGL-1 whose roles is to prevent oedema. Besides, other components such as glutathione and allopurinol, raffinose, and lactobionate are present in these solutions [8].

Several clinical trials and experimental investigations have been carried out to compare the benefits of all preservation solutions [5,9,10,11,12,13]; but the analysis comparing IGL-1 versus HTK are poor [5,11], especially when they deal with the underlying protection mechanisms responsible for the safety of fatty liver graft against cold ischemia reperfusion injury. Recent HTK limitations in liver transplantation have been pointed out from the European Transplantation Register [5]. In accordance with this, Meine and cols have recently reported that the use of HTK in liver transplantation trials evidenced higher ischemia-reperfusion damage during the first postoperative days than IGL-1 solution [11]. In a previous work we demonstrated the relevance of AMP-activated protein kinase (AMPK) in fatty liver preservation when IGL-1 was used and compared to HTK [14]. 

With this in mind, we have explored the underlying mechanisms responsible for graft protection using IGL-1 and HTK preservation solutions, given that they have not been fully investigated. Data reported here, demonstrates for the first time the relevance of mTOR-autophagy system involved in fatty liver preservation, especially when IGL-1 and HTK preservation solutions are used.

## 2. Results

First, we aimed to investigate the effect of both preservation solutions IGL-1 and HTK on hepatic injury in obese rats (Table 1). Fatty livers cold preserved in IGL-1 or HTK were associated with significant rises in AST and ALT levels in perfusate. However, when comparing both preservation solutions, IGL-1 increase was minor and not significant versus the control animals. As well, the same results were obtained when measuring GLDH activity.

**Figure 1:**
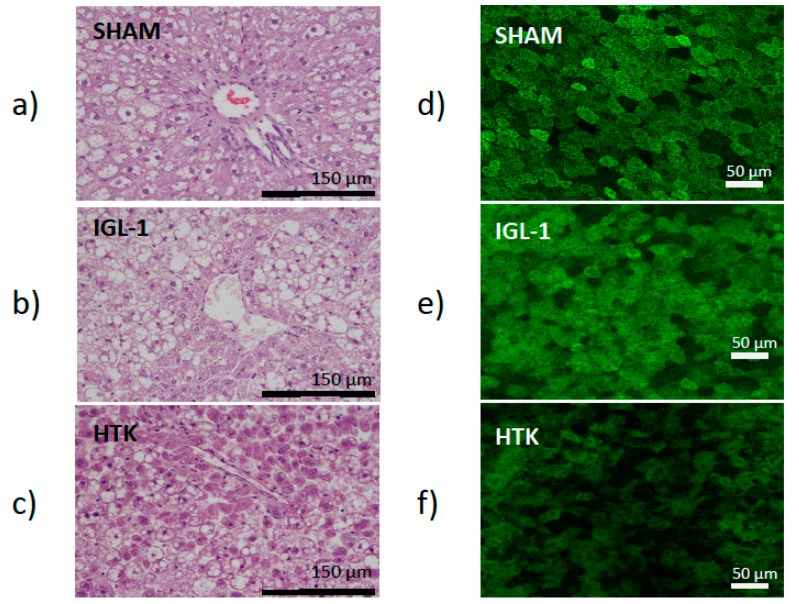
Effects of IGL-1 and HTK preservation solutions on histological changes in fatty liver grafts in cold storage. Left panel: Representative images of hematoxylin-eosin stained liver sections for each experimental group were taken with 20× lens. After hematoxylin-eosin staining ((**a**) SHAM, (**b**) Institute Goeorges Lopez 1 (IGL-1), (**c**) Histidine-Tryptophan-Ketoglutarate (HTK)), samples were analyzed in a scale from 0 (no damage) to 4 (maximum damage). While control group average damage was considered 0, IGL-1 group was 1.67 and HTK 3.06. Right panel: Representative images of confocal microscopy findings on hepatic sections for each experimental group ((**d**) SHAM, (**e**) IGL-1, (**f**) HTK).

This was consistent with the histological (liver injury) and confocal microscopy findings (mitochondrial damage). Liver injury was evaluated by histology using hematoxylin/eosin staining. As indicated in Figure 1a–c (left panel), the IGL-1 conserved livers were more preserved than those conserved in HTK which revealed cell swelling and dissociation with liver architecture destruction. By contrast, histological evaluation of the controls animals showed normal hepatic architecture with macro- and micro-vesicular fatty infiltration. Moreover, this higher prevention of hepatic injury when the livers were preserved in IGL-1 was accompanied by a better protection of liver mitochondrial damage, as revealed the confocal mitochondrial findings in Figure 1d–f (right panel) where changes in mitochondrial membrane potential were determined with rhodamine 123 staining. In SHAM livers where there is no damage, Rhodamine 123 labelling (green) concentrates in healthy polarized mitochondria network providing a highly contrasted image, whereas as ischemic damage increases, mitochondria lose their membrane potential and therefore Rhodamine 123 labelling is diffused. In this case, the mitochondrial depolarization was evidenced by a green color intensity diminution in livers preserved in HTK solution vs the ones preserved in IGL-1 solution and controls, respectively. 

**Figure 2:**
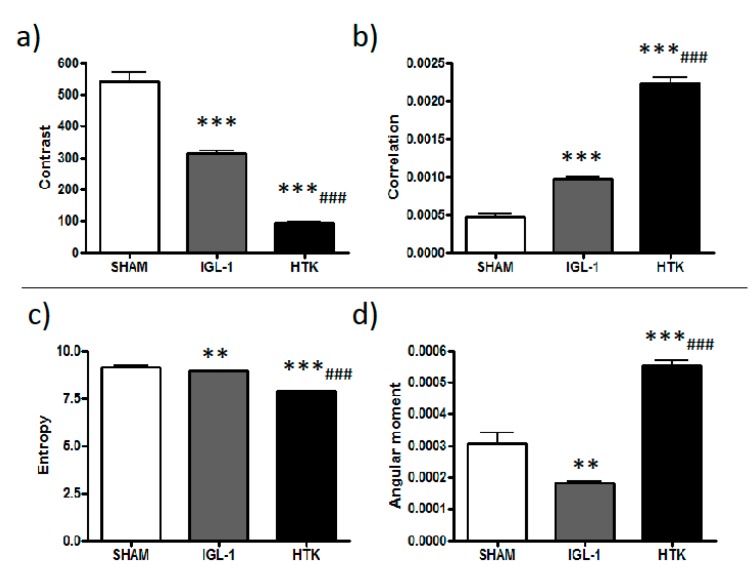
Parameters obtained after a co-occurrence matrix analysis of confocal microscopy images (Figure 1d (SHAM), Figure 1e (IGL-1), Figure 1f (HTK)). Bar graphs represents contrast (**a**), correlation (**b**), entropy (**c**) and Angular moment (**d**) mean ±SD, ** *p* < 0.005 vs. SHAM, *** *p* < 0.0005 vs. SHAM, ^###^ *p* < 0.0005 vs. IGL-1.

In order to quantitatively distinguish Rhodamine 123 labelling patterns, concentrated (membrane potential preserved) versus the diffused ones (lose of membrane potential), texture analysis was applied. Texture analysis are mathematical measurements that describe the distribution and relationship between pixel intensities. Parameters such as contrast, angular second moment, correlation or entropy are parameters used to express the homogeneity and complexity of an image in a co-occurrence matrix analysis. In this sense, data reported in Figure 2 contributes to better explain the changes observed on mitochondrial potential membrane depolarization in the livers preserved in IGL-1 and HTK solutions vs control (see Figure 1d–f). According to our data, there is a significant difference between all groups, suggesting that during cold ischemia, mitochondrial membrane potential is better preserved when IGL-1 solution is used compared with HTK. 

**Figure 3:**
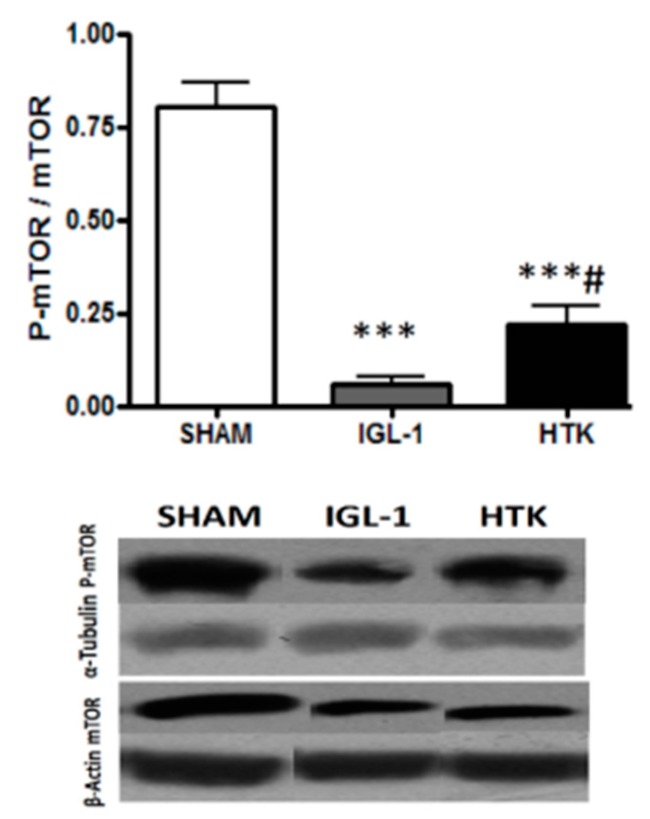
p-mTOR/mTOR expression in fatty liver grafts preserved in HTK or IGL-1 solutions. The ratio of p-mTOR to total mTOR was quantified by densitometry based on immunoblot images. Bar graphs represent the mean ±SD. *** *p* < 0.0005 vs. SHAM, ^#^ *p* < 0.05 vs. IGL-1.

The kinase mTOR plays an important role in in growth and metabolism and controls the progress of autophagy, being inhibited when autophagy is induced [15,16]. Therefore we evaluated the changes in mTOR system in fatty liver grafts preserved during 24 h at 4 °C in IGL-1 or HTK solutions. As shown in Figure 3, a significant diminution of p-mTOR/mTOR ratio revealed that mTOR was significantly more inhibited in livers preserved in IGL-1 rather than in those preserved in HTK.

**Figure 4:**
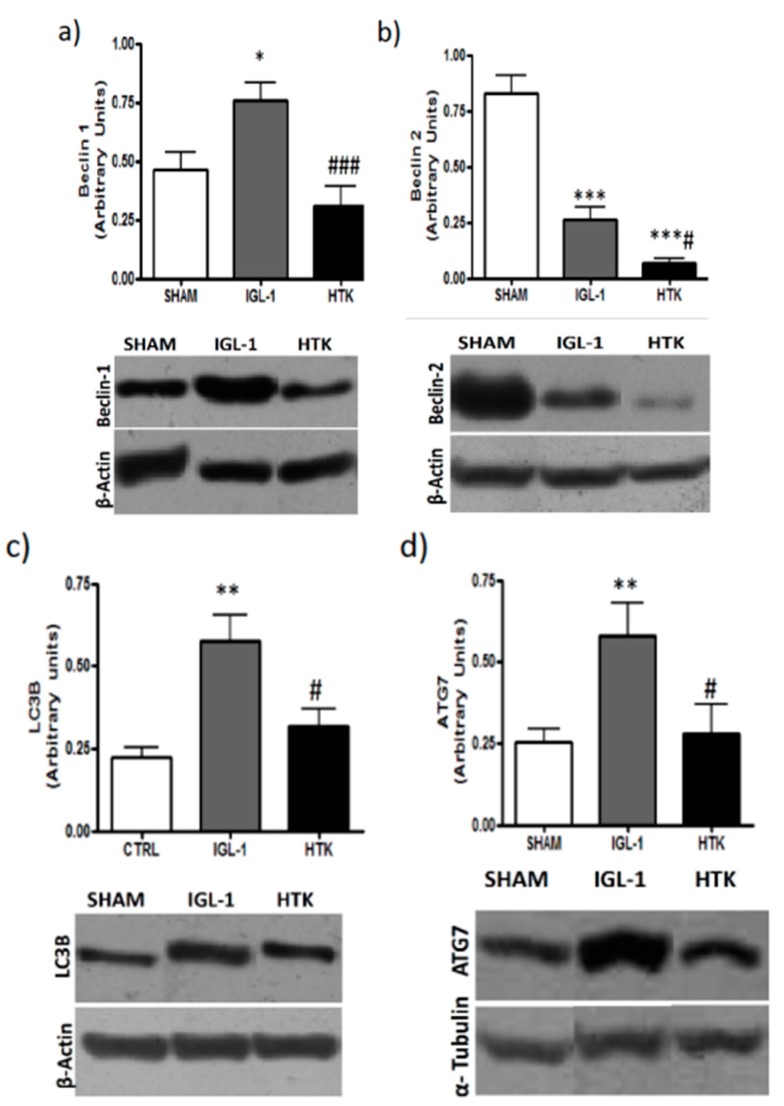
Liver autophagy in fatty liver grafts stored in IGL-1 and HTK vs. controls (24 h; 4 °C) measured as Beclin 1 (**a**), Beclin 2 (**b**), LC3B (**c**) and ATG7 (**d**). Bar graphs represent the mean ±SD. * *p* < 0.05 vs. SHAM, ** *p* < 0.005 vs. SHAM, *** *p* < 0.0005 vs. SHAM, ^#^ *p* < 0.05 vs. IGL-1, ^###^ *p* < 0.0005 vs. IGL-1.

Given that mTOR has been postulated as an important mediator of autophagy [16], we evaluated whether the lessened levels of the active isoform of mTOR in the livers preserved in IGL-1 solution could correlate with higher induced autophagy by measuring well-known markers such as Beclin-1, Beclin-2, LC3B and ATG7. Figure 4 shows higher increases in Beclin-1, Beclin-2, LC3B and ATG7 expression levels in those livers preserved in IGL-1 when compared to HTK, evidencing the existence of an increased autophagy process during cold preservation in IGL-1 solution. 

**Figure 5:**
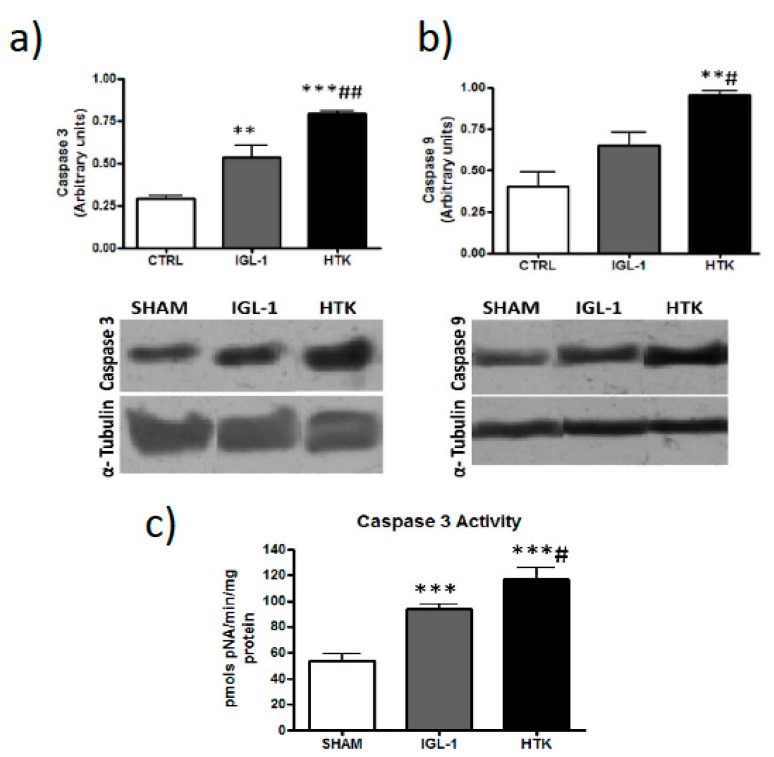
Apoptosis (measured as cleaved caspase 3 (**a**), caspase 9 (**b**) and caspase 3 activity (**c**)) in liver grafts preserved in IGL-1 and HTK vs controls. Bar graphs represent the mean ±SD. ** *p* < 0.005 vs. SHAM, *** *p* < 0.0005 vs. SHAM, ^#^ *p* < 0.05 vs. IGL-1, ^##^ *p* < 0.005 vs. IGL-1.

**Figure 6:**
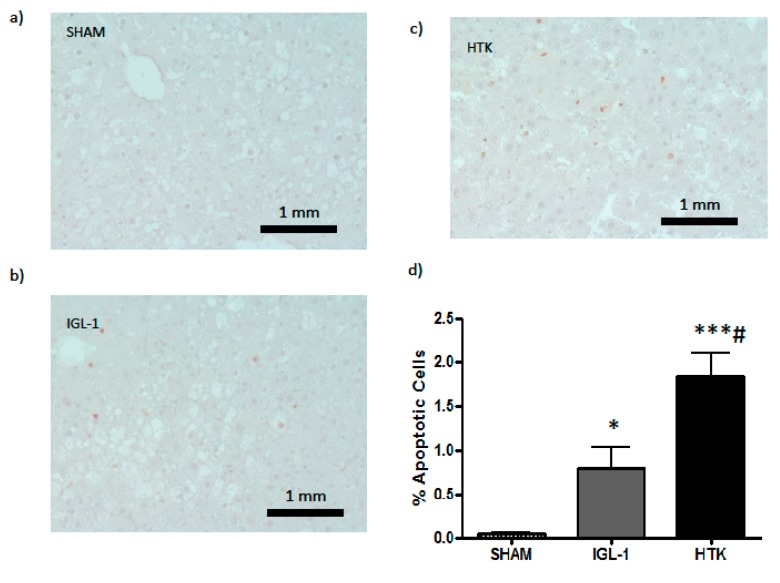
Apoptotic hepatic cells detected with TUNEL assay. (**a**) SHAM, (**b**) IGL-1, and (**c**) HTK. (**d**) Apoptotic cells (stained with red) were counted and damage was assessed as a percentage of positive cells for each group. Bar graphs represent the mean ±SD. * *p* < 0.05 vs. SHAM, *** *p* < 0.0005 vs. SHAM, ^#^ *p* < 0.05 vs. IGL-1.

Following that hypothesis, we assessed apoptosis because it is well known that is affected by cold ischemic time (24 h at 4 °C, in our experimental conditions) [17]. Data reported in Figure 5 and Figure 6 revealed that ILG-1 cold-preserved livers were associated with more pronounced reduced levels of apoptosis compared to HTK solution.

**Figure 7:**
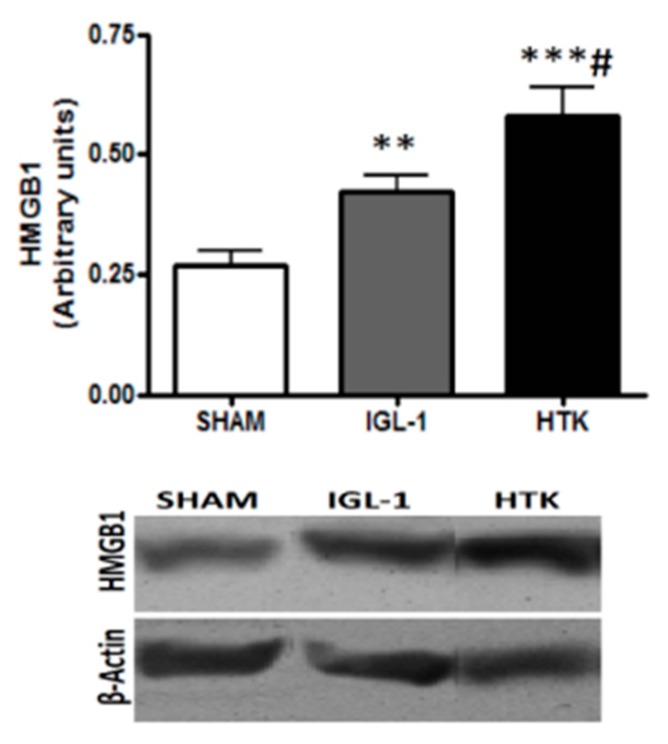
Changes in HMGB1 expression in controls and livers preserved in IGL-1 or HTK. Bar graphs represent the mean ±SD, ** *p* < 0.005 vs. SHAM, *** *p* < 0.0005 vs. SHAM, ^#^ *p* < 0.05 vs. IGL-1.

In addition, we determined the expression of High Mobility Group Box 1 (HMGB1) in preserved liver grafts (Figure 7), which is considered an alarm signal as a response against cold ischemic injury [18]. As shown in Figure 6, the grafts preserved in IGL-1 solution showed lessened levels in HMGB1 expression than those conserved in HTK. These findings indicate that IGL-1 exerted a substantial protective effect by abrogating the liver injury subsequent to cold ischemia.

## 3. Discussion

Extensive research has focused on the optimization of organ preservative solutions. In the present study, we compared the efficacy of HTK and IGL-1 solution, and we have found that IGL-1 preservation solution is more effective in preventing rat liver damage after 24 h of cold preservation than HTK solution.

Histological and confocal microscopy findings confirmed the lessened damage in livers stored in IGL-1 cold when compared to those preserved with HTK. This was concomitant with prevention of graft liver mitochondrial status after 24 h of cold ischemia, as evidenced by the texture analysis and histological techniques. This better liver graft protection during cold storage in IGL-1 vs. HTK solutions was closely associated with relevant changes in the p-mTOR/mTOR ratio. 

mTOR is an evolutionary conserved serine/threonine protein kinase held responsible of cell growth, proliferation and metabolism through its phosphorylation [15]. Under ischemic conditions (deprivation of energy), alternative ways to acquire energy are needed, and autophagy process is believed to enable cell survival through recycling of cell metabolites [19]. In this sense, inhibition of autophagy is harmful for cell survival, and p-mTOR is a known autophagy inhibitor [20] especially in fatty livers [21]. 

In our experimental data we evidenced higher levels of p-mTOR in livers preserved with HTK than in the ones preserved with IGL-1, therefore decreasing cyto-protective autophagy and leading to more cell death as outcome.

It is well known that autophagy is a tightly regulated, highly conserved physiologic process that involves recycling of cytoplasmic proteins and occurs at low-baseline levels in all cells and in energy depletion conditions [22,23]. Our results evidenced the existence of a “cyto-protective autophagy” process induced during liver graft cold preservation which was more important in liver preserved in IGL-1 than HTK. This was confirmed by the higher levels of Beclin-1, Beclin-2, LC3B and ATG7 expression in livers preserved in IGL-1 than in the ones preserved with HTK. It is important to remark that under these experimental conditions of liver cold storage, the process is protective because is not excessive enough in time and strength to cause cell death, as revealed by the reduced levels of apoptosis. In this long ischemic preservation conditions, autophagy is preventive against organ damage, while increased apoptosis levels leads to a future organ failure. On the other hand, this contrasts with the autophagy that is responsible for the cell death during reperfusion [24] and the further compromise of the graft viability. In this sense, the prevention of HMGB1, an inflammatory cytokine and an alarm signal for cold ischemic injury, could contribute to prevent the exacerbated microcirculatory events present when fatty liver grafts are vascularized. 

**Scheme 1:**
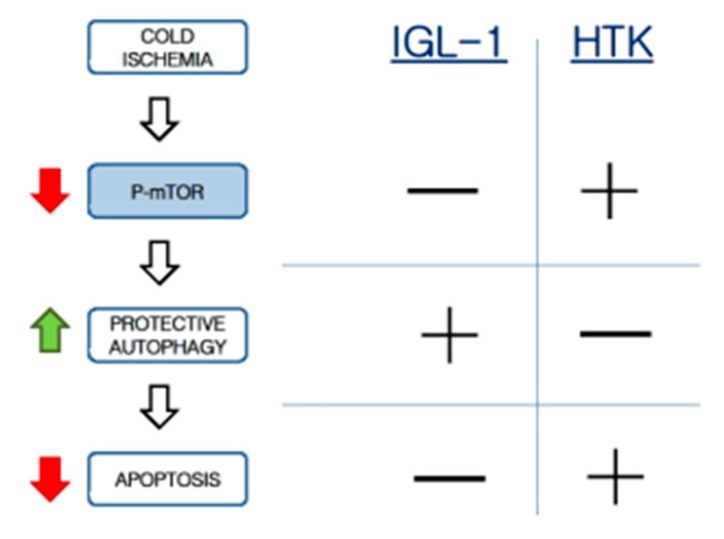
Suggested protective pathway involved in fatty liver preservation. Increases are represented by green arrows, while decreases are represented with red arrows (left side). On the right side – and + represents the behavior of the livers with the mentioned solution regarding the factor described on the left side of the scheme.

Data reported here are consistent with previously ones published by our group when IGL-1 and HTK were compared. Under the same conditions as this present study, AMP-activated protein kinase (AMPK) levels were higher in its active isoform (pAMPK) when IGL-1 solution was used [14]. Bibliography reports the existence of the AMPK/mTOR/autophagy axis [25] which has never been demonstrated before under our experimental conditions, and it takes special relevance as a possible protective target for liver graft conditioning during organ preservation, in order to limit organ injury and to maintain the graft quality before transplantation. With this in mind, we could hypothesize that this AMPK/mTOR/autophagy axis might be working here too, and IGL-1 solution could act as a “preconditioning solution” for the preservation of liver grafts, as similarly occurred for the liver ischemic preconditioning [26,27,28,29]. In this case, the protective mechanisms during cold ischemia would be associated with AMPK activation, mTOR inhibiton, autophagy induction, and apoptosis prevention. Consequently, the mTOR system can be considered one of the main actors in this pathway to induce cytoprotective autophagy and prevent hepatic apoptosis during the cold static preservation stage (Scheme 1).

## 4. Materials and Methods

### 4.1. Animals

Male obese Zucker rats (11 weeks aged) were housed in conventional animal facilities where temperature and humidity were controlled with a 12 h light/dark cycle. Animals had free access to water and standard diet. All experiments were conducted according to the Ethics Committees for Animal Experimentation (CEEA, Directive 483/16), University of Barcelona, following European Union Regulations for animal experiments (EU guideline 86/609/EEC) and in accordance with protocols approved on 14 July 2016 (NO. 483116). 

### 4.2. Liver Procurement and Experimental Groups

The surgical procedures were carried out as described elsewhere [14]. After cannulation of the common bile duct, the portal vein was isolated and the splenic and gastroduodenal veins were ligated. All animals were randomly distributed into different experimental groups, as follows:

*SHAM Group* (*n* = 6). Male obese Zücker rats were subjected to transverse laparotomy as follows: we carried out ligatures at right suprarenal vein, diaphragmatic vein and hepatic artery levels. Following, livers were rinsed through the portal vein with Ringer lactate solution (200 mL) and immediately stored at −80 °C for subsequent analyses.

*IGL-1 group* (*n* = 6). Steatotic liver grafts were stored in IGL-1 solution at 4 °C for 24 h and immediately washed with Ringers solution. Effluents and tissue specimens were collected at −80 °C for subsequent analyses.

*HTK group* (*n* = 6). Steatotic liver grafts were stored in HTK solution at 4 °C for 24 h and immediately washed with Ringers solution. Effluents and tissue specimens were stored at −80 °C for subsequent analyses. 

### 4.3. Biochemical Measurements

#### 4.3.1. Liver Injury 

Hepatic injury was evaluated by alanine aminotransferase (ALT) and aspartate aminotransferase (AST) levels using commercial kits from RAL (Barcelona, Spain), as previously reported [26]. Briefly, 100 μL of effluent washout liquid or perfusate was added to 1 mL of the substrate provided by the commercial kit, and then transaminase activity was measured at 340 nm with a UV spectrometer and calculated following the supplier’s instructions. Results were normalized using a commercial calibrator Biocal, RAL (Barcelona, Spain).

#### 4.3.2. Mitochondrial Damage 

Glutamate dehydrogenase (GLDH) in serum levels is an indicative marker of mitochondrial damage. GLDH levels in liver perfusate after 24 h cold ischemia were measured using a commercial kit from Randox Lab (Crumling, UK) by quantifying the decrease of absorbance at 340 nm according manufacturer’s protocol [8].

#### 4.3.3. Caspase activity

Caspase activity was measured in tissue homogenate using a commercial kit from Sigma-Aldrich (ref. CASP3C-1KT) according to the manufacturer’s instructions.

### 4.4. Histopathological Examination 

Liver samples were fixed in 10% neutral buffered formalin and embedded in Paraplast, and 5-µm sections were stained with hematoxilin and eosin according to standard procedures. Histologic evaluation was graded semiquantitatively from 0 (no damage) to 4 (severe cellular damage, such as vacuolization, cell dissociation, cell swelling, and disintegration of hepatic architecture). Images were taken with 20× objective.

### 4.5. Confocal Microscopy 

After 24 h of hypothermic preservation, fatty livers were then carefully sectioned (0.5 cm^3^ fragments) and the internal side of the liver was exposed on the glass coverslip mounted on the stage of a Leica TCS SP5 resonant scan multiphoton confocal microscope (Leica Microsystems Heidelberg GmbH, Heidelberg, Germany) equipped with a HCX IR APO L 25× water immersion objective (Numerical Aperture 0.95), scanner at 400 lines/s, and a near infrared Titanium:Saphire laser (MaiTai, SpectraPhysics, Santa Clara, CA, USA) for two-photon excitation running at 800 nm. Two-photon excitation was performed at 800 nm and emission of the different fluorescent dyes was captured at the range for rhodamine 123 (500–550 nm) [30,31].

#### Co-Occurrence Matrix Analysis

In order to quantitatively distinguish Rhodamine 123 labelling patterns, concentrated (membrane potential preserved) versus the diffused ones (loss of membrane potential), texture analysis was applied. Texture analysis are mathematical measurements that describe the distribution and relationship between pixel intensities. 

In this study texture measurements are based on Gray-level co-occurrence Matrix (GLCM) that is a second-order statistical method, which measures texture features on the basis of the relationship between 2 pixels. There are several quantitative parameters as exposed in Figure 2, that describe the texture of a 2D image [32]. Texture analysis has been proved successful for medical purposes [33].

In our case, for each sample, 40 fields were analyzed (a total surface of 1.90 mm × 3 mm). Results interpretation goes as it follows:

*Contrast*: Contrast parameter provides information on the intensity difference between neighbor pixels. As SHAM animals have no damage, mitochondrial membrane potential will remain high, and therefore labelling is concentrated in mitochondria showing a highly contrasted mitochondria network. In conditions of ischemic damage where mitochondria polarity is lost, labelling from mitochondria is diffused providing an image more uniform and with lower contrast.

*Correlation*: It measures linear dependency of gray levels of neighboring pixels. It can be understood as a uniformity value in neighboring pixels. Correlation is higher in HTK where labelling is more diffused than in IGL-1 and SHAM condition. High correlation comes from the fact that diffused labelling has a bigger number of pixels with similar values of intensity that co-variate. 

*Entropy*: Entropy is a measure related to orderliness, how regular the pixel value differences are. Entropy decreases as pixel values differences are more regular. It describes the randomness and order of the sample. The higher the randomness, the higher the entropy is. In SHAM animals, entropy is lower due to labelling more concentrated in mitochondria, whereas as labelling is more diffused, randomness and entropy increase. Increase in entropy in HTK compared to IGL-1 describes a higher diffusion of the dye and therefore loss in membrane potential.

*Angular second moment*: It is also related to orderliness and it measures image homogeneity of greys. In HTK samples labelling has diffused and it is very homogeneous, so angular second moment in HTK is higher compared to IGL and SHAM.

All texture parameters describe how labelling is being distributed differently in all conditions. In all parameters, IGL-1 values are more similar to SHAM model indicating that under preservation conditions with IGL-1 a higher number of hepatocytes keep mitochondria polarized. In order to simplify description of microscopy results we didn’t include the explanation of the individual mathematical texture parameters.

### 4.6. TUNEL Assay

DNA fragmentation was determined using a TUNEL assay kit according to the manufacturer’s instructions (Apoptag Peroxidase In Situ Apoptosis Detection Kit, Merk, Darmstadt, Germany). Hepatic cells were analysed in 3 different tissue sections per animal, and a total of 10 randomized HPF per animal were counted (400 hepatic cells per field). The ratio of positive cells to the total number was calculated.

### 4.7. Western Blotting 

Separated on 6–15% sodium dodecyl sulfate polyacrylamide gel electrophoresis (SDS-PAGE) gels. Proteins were blotted into poly-vinylidene fluoride (PVDF) membranes (Biorad, Madrid, Spain) and immunoblotted overnight at 4 °C with antibodies directed against the following proteins: phosphorylated mTOR and ATG7 (Cell Signaling Technology Inc., Beverly, MA, USA), cleaved caspase 3, cleaved caspase 9, mTOR, Beclin-1, Beclin-2, (Santa Cruz Biotechnology, Santa Cruz, CA, USA), LC3B (Abcam Inc., Cambridge, UK), β-actin (Sigma Chemical, St. Louis, MO, USA) and HMGB1 and α-tubulin (Abcam, Cambridge, UK). After washing, membranes were incubated with appropriate peroxidase-conjugated secondary antibody for 1 h. Immuno-labeled bands were detected using Western-Bright ECL-HRP Substrate (Advansta, Menlo Park, CA, USA) and quantified using the Quantity One software for image analysis. Results were expressed as densitometry ratios between the protein of interest and the loading control (β-actin).

### 4.8. Statistical Analysis

Data are expressed as means ± SE and compared statistically by variance analysis, followed by the Student-Newman Keuls test using GraphPad Prism version 4.02 for Windows (GraphPad Prism software; Accession data: 17 May 2004). *p* < 0.05 was considered significant.

## Figures and Tables

**Table 1:** Liver injury (measured as AST and ALT) and mitochondrial damage (measured as GLDH).

Biomarker	SHAM	IGL-1	HTK
AST (U/I)	29 ± 12.8	71 ± 37 *	163 ± 78 **^##^
ALT (U/I)	35 ± 12	67 ± 26 *	131 ± 40 *^#^
GLDH (U/I)	6.2 ± 3.8	31.5 ± 20.9 *	100.1 ± 53 **^##^

Significance is expressed as: * *p* < 0.05 vs. SHAM, ** *p* < 0.005 vs. SHAM, ^#^ *p* < 0.05 vs. IGL-1, ^##^ *p* < 0.005 vs. IGL-1. IGL-1: Institute Goeorges Lopez 1; HTK: Histidine-Tryptophan-Ketoglutarate; AST: Aspartate aminotransferase; ALT: Alanine aminotransferase; GLDH: Glutamate dehydrogenase.
